# Characterization of p190-Bcr-Abl chronic myeloid leukemia reveals specific signaling pathways and therapeutic targets

**DOI:** 10.1038/s41375-020-01082-4

**Published:** 2020-11-09

**Authors:** Shady Adnan-Awad, Daehong Kim, Helena Hohtari, Komal Kumar Javarappa, Tania Brandstoetter, Isabella Mayer, Swapnil Potdar, Caroline A. Heckman, Soili Kytölä, Kimmo Porkka, Eszter Doma, Veronika Sexl, Matti Kankainen, Satu Mustjoki

**Affiliations:** 1 Hematology Research Unit Helsinki, University of Helsinki and Helsinki University Hospital Comprehensive Cancer Center, Helsinki, Finland; 2 Translational Immunology Research Program and Department of Clinical Chemistry and Hematology, University of Helsinki, Helsinki, Finland; 3 Clinical Pathology Department, National Cancer Institute, Cairo University, Cairo, Egypt; 4 Institute for Molecular Medicine Finland (FIMM), Helsinki Institute for Life Science, University of Helsinki, Helsinki, Finland; 5 Institute of Pharmacology and Toxicology, University of Veterinary Medicine Vienna, Vienna, Austria; 6 iCAN Digital Precision Cancer Medicine Flagship, Helsinki, Finland; 7 HUS Diagnostic Center, HUSLAB, Helsinki University Hospital, Helsinki, Finland

**Keywords:** Cancer genomics, Oncogenesis, Translational research, Chronic myeloid leukaemia

## Abstract

The oncogenic protein Bcr-Abl has two major isoforms, p190^Bcr-Abl^ and p210^Bcr-Abl^. While p210^Bcr-Abl^ is the hallmark of chronic myeloid leukemia (CML), p190^Bcr-Abl^ occurs in the majority of Philadelphia-positive acute lymphoblastic leukemia (Ph + ALL) patients. In CML, p190^Bcr-Abl^ occurs in a minority of patients associating with distinct hematological features and inferior outcomes, yet the pathogenic role of p190^Bcr-Abl^ and potential targeting therapies are largely uncharacterized. We employed next generation sequencing, phospho-proteomic profiling, and drug sensitivity testing to characterize p190^Bcr-Abl^ in CML and hematopoietic progenitor cell line models (Ba/f3 and HPC-LSK). p190^Bcr-Abl^ CML patients demonstrated poor response to imatinib and frequent mutations in epigenetic modifiers genes. In contrast with p210^Bcr-Abl^, p190^Bcr-Abl^ exhibited specific transcriptional upregulation of interferon, interleukin-1 receptor, and P53 signaling pathways, associated with hyperphosphorylation of relevant signaling molecules including JAK1/STAT1 and PAK1 in addition to Src hyperphosphorylation. Comparable to p190^Bcr-Abl^ CML patients, p190^Bcr-Abl^ cell lines demonstrated similar transcriptional and phospho-signaling signatures. With the drug sensitivity screening we identified targeted drugs with specific activity in p190^Bcr-Abl^ cell lines including IAP-, PAK1-, and Src inhibitors and glucocorticoids. Our results provide novel insights into the mechanisms underlying the distinct features of p190^Bcr-Abl^ CML and promising therapeutic targets for this high-risk patient group.

## Introduction

Bcr-Abl is a hybrid oncokinase protein, which is expressed upon t(9;22) reciprocal translocation resulting in fusion between *BCR* and *ABL1* genes [1]. Depending on the breakpoint within the *BCR* partner, different hybrid genes encoding distinct Bcr-Abl isoforms are formed [2]. The most commonly occurring Bcr-Abl isoforms are designated as p190^Bcr-Abl^ (also known as p185^Bcr-Abl^) and p210^Bcr-Abl^ (referred to as p190 and p210 hereafter), based on apparent molecular weights [3]. Except for the lack of Dbl homology and Pleckstrin homology domains in p190, both isoforms share the same sequence and protein domains [4, 5]. RAG activity has been suggested to contribute to p190 initiation, however the mechanisms underlying different isoform formation are largely unknown [6]. The p190 and p210 isoforms associate with distinct disease phenotypes. Being expressed in about 95% of patients, p210 is the hallmark of chronic myeloid leukemia (CML). In contrast, p190 is expressed in approximately three-fourths of Philadelphia-positive acute lymphoblastic leukemia (Ph + ALL) patients, while the remaining one-fourth of patients express the p210 isoform [7–9].

In CML, p190 occurs as the sole Bcr-Abl isoform at diagnosis in a minority of patients (1–2%) [10, 11] and is coexpressed with p210 in about 5-7% of patients [12]. CML patients solely expressing p190 have shown to exhibit characteristic hematological features including monocytosis and frequent additional chromosomal abnormalities [13, 14]. In addition, p190 is associated with increased risk of progression to blast phase specially of lymphoid phenotype and inferior response to the standard tyrosine kinase inhibitor (TKI) treatment [10, 15]. The underlying transcriptional programming and signaling pathways implicated in p190-CML pathogenesis are largely unknown. Given the inferior responses of p190 CML, there is a need for effective novel drug candidates.

Various models have been developed to investigate leukemogenic potential of different Bcr-Abl isoforms [16–18]. In mouse models, the expression of p210 has been associated with slowly progressive CML-like disease while p190 is associated with short latency and acute B-cell leukemia, suggesting the contribution of Bcr-Abl isoforms to specific fates [18, 19]. Despite several studies have investigated the transcriptional activity and downstream signaling of Bcr-Abl, only a few studies have systematically compared the differences between p190 and p210 isoforms in uniform models. Earlier studies have focused on selected signaling molecules, namely JAK-STAT, and revealed comparable phosphorylation activities of both isoforms with few isoform-specific hits [20]. Generally, p190 has shown to exhibit a higher kinase activity and increased autophosphorylation [18, 21, 22]. Recent studies have investigated the global interactome and signaling profiles of p190 and p210 in a uniform mouse cell line background, and demonstrated differences in isoform-specific interactome and signaling pathways [23, 24]. To date no study has directly compared p190 and p210 in primary patient cells, especially in CML. Furthermore, the drug sensitivity differences between both isoforms have not been systematically studied either with cell line models or primary cells of CML and Ph+ALL.

In order to explore the unique pathways involved in p190-CML leukemogenesis, we integrated exome sequencing, RNA sequencing and phosphorylation profiling to characterize a small cohort of p190-CML patients. We validated our findings by directly comparing the gene expression and phosphorylation profiles between p190 and p210 in two different hematopoietic progenitor cell lines (Ba/f3 and HPC-LSK). Furthermore, we used a comprehensive drug testing to investigate isoform-specific sensitivities and identified promising targeted therapies. Our study provides the first consolidated view of the molecular pathogenesis of p190-CML and identifies promising drug candidates which can be translated into tailored treatment strategies for these high-risk patients.

## Materials and methods

### Patients

Clinical and hematological data of p190-CML patients (*n* = 4), p210-CML patients (*n* = 3), and Ph+ALL patients (*n* = 10) are summarized in Supplementary Table 1. All patients were recruited from Helsinki University Hospital (HUH), Finland. Written informed consents were obtained from all patients in accordance with the declaration of Helsinki.

### Cell lines

Mouse Ba/f3 cell line retrovirally transfected with BCR-ABL1-GFP encoding either p190/p210 was a kind gift from Prof. Nikolas von Bubnoff (Universitätsklinikum Freiburg, Germany). HPC-LSK progenitor cell lines [25] were generated in Prof. Veronika Sexl lab (University of Veterinary Medicine of Vienna, Austria), and it was similarly retrovirally transfected with respective GFP-marked transcript. In both p210-Ba/f3 and p210-HPC-LSK cell lines, cells were expressing e14a2 (b3a2) transcript. Cytokine-independent transduced Ba/f3 cells were grown in RPMI-1640 media (Lonza), supplemented with 10% FBS, 2 mM L-glutamine (Lonza) and 100 U/mL penicillin and 100 μg/mL streptomycin (Gibco). HPC-LSK cells were cultured in IMDM media (Sigma), supplemented with 5% FBS, 0.75 × 10^−4^ M 1-Thioglycerol (Sigma), 12.5 ng/ml recombinant human IL6 (Peprotech), 2 mM L-glutamin, and antibiotics as indicated for Ba/f3 cells (for both parental and transduced lines) in addition to inhouse-prepared stem cell factor (only for parental line).

### Whole-exome sequencing (WES), RNA sequencing, and data analysis

Genomic DNA was extracted from diagnostic PMNCs samples of four p190-CML patients. Processing of WES samples was performed as previously described [26] using HiSeq instrument (Illumina) according to the manufacturer’s protocols. For RNA sequencing, total RNA was extracted from patient samples (6 CML) in addition to cell line samples. For each cell line, three biological replicates were used for either p190, p210, and parental conditions. RNA isolation and further processing were performed as earlier described [26]. WES and RNA-sequencing workflow, bioinformatic processing, and adjustment for possible batches and confounding factors are detailed in Supplementary materials.

### Drug sensitivity and resistance testing (DSRT)

To screen the drug sensitivity profile of Ba/f3 cell lines, a comprehensive oncology drug library consisting of 156 approved drugs and 372 preclinical and investigational compounds was used. For the less proliferating HPC-LSK cells, a custom library of selected 65 compounds were designed and applied (Supplementary Table 2). Drugs were tested in five concentrations covering a 10,000-fold range. DSRT and quantification of drug sensitivity scores were performed as earlier described [27]. For both cell lines, three independent replicates of each isoform setting were profiled. Details of DSRT workflow are described in Supplementary materials.

### Phosphoproteomic profiling

For profiling the phosphorylation activity of p190 and p210 isoforms in cell line models, a Tyrosine Phosphorylation Proarray (Full Moon Biosystems, #PST228) featuring 228 phospho-tyrosine sites was used. Sample preparation and processing were performed according to manufacturer’ instructions. Array scanning and image analysis were performed by Full Moon Bioscience and data were normalized to the median antibody signaling value. Western blotting was used to validate findings in cell lines and CML patient samples (three p190 and three p210). For Ph+ALL samples, phosphorylation of selected proteins was investigated by flowcytometry using iQue Screener Plus flow cytometer (Intellicyt). Further details are described in Supplementary materials.

### Colony forming assay

For HPC-LSK cell lines, 2.5 × 10^3^ cells were re-suspended in 0.4 ml IMDM culture medium supplemented by 2% FBS and the samples were mixed with 4 ml of methylcellulose (MethoCult™ M3231, STEMCELL Technologies) without adding cytokines. Drugs (imatinib, dasatinib, LCL161, FRAX486, dexamethazone, idasanutlin) and recombinant mouse IFN-alpha (eBioscience, #14-8312-80) were added in the indicated concentrations. 1.1 ml were plated on 35–mm-dishes in triplicate according to instructions of the manufacturer. Colonies were counted after 14 days using an inverted microscope. For phenotyping of the clones, cells were harvested from colonies at day 14 and stained with a panel of antimouse antibodies including CD19-PE (eBioscience, #12-0193-82), CD11b-PerCP-Cy™5.5 (#561114), CD45R/B220-BV421 (#562922) from BD Bioscience and CD3-APC (#100236), CD45-APC-Cy7 (# 557659) from Biolegend. Cells were acquired by FACSVerse (BD Biosciences).

### Statistical testing

Mann–Whitney U-test, Two-tailed Student *t* test, Fisher Exact test, Pearson’ correlation, and Spearman correlation tests were computed using GraphPad Prism 8 software or R 3.5.0.

## Results

### p190-CML patients demonstrate distinct hematological features, inferior responses to imatinib and abundance of epigenetic modifier mutations

**Fig. 1:**
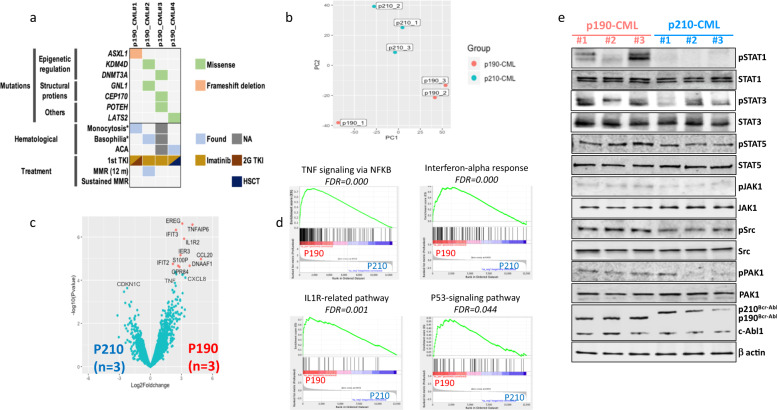
Clinical, genomic and signaling profiles of p190-CML patients. **a** Landscape of nonsilent mutations identified by WES from p190-CML patients (*n* = 4). Variants that has been linked with cancer (COSMIC database) are shown in the figure. Complete list of mutations can be found from Supplementary Table 3. The bottom tracks show clinical (monocytosis (>10% of total leukocytic count), basophilia, additional chromosomal abnormalities (ACAs)) and treatment features of patients (type of treatment, achievement of major molecular response (MMR) at 12 months, sustained MMR). The color of the variant box indicates the type of mutation. **b** PCA analysis of transcriptional data (protein coding genes) from CML patients. **c** Volcano plot of protein coding genes between p190-CML (*n* = 3, right) and p210-CML (*n* = 3, left). Each gene is represented by a black dot and significant differentially expressed genes (*Q* < 0.05, Bayesian statistical test) are colored red. **d** Gene set enrichment analysis (GSEA) output showing upregulation of TNF, IFNα, IL1R, and P53 signaling pathways in p190-CML compared to p210-CML patients. **e** Western blot analysis of JAK/STAT pathway, Src and PAK1 signaling molecules in CML patients. For each protein a phospho specific antibody was used in addition to an antibody against the protein to assess the overall protein expression.

To explore the molecular pathogenesis of p190-CML, we performed genetic profiling of diagnostic samples of four p190-CML patients with a median age of 72.5 years (range, 50–80) (Fig. 1a, Supplementary Table 1). All patients received imatinib as a frontline treatment. Except for one patient who achieved a fluctuating molecular response, the rest of patients showed primary resistance to imatinib. Patients were then shifted to a 2nd generation TKI, nilotinib, achieving minimal responses, or proceeded to stem cell transplantation (allo-SCT) (Fig. 1a, Supplementary Table 1). Using WES, we identified 46 variants in p190-CML samples (median per patient = 9.5, range: 4–26), including potentially relevant variants in *ASXL1, DNMT3A*, and *KDM4D* epigenetic modifier genes (Fig. 1a, Supplementary Table 3).

### Transcriptional and phosphorylation analysis reveals activation of IFN/JAK1/STAT1 axis in p190-CML patients

Next, we investigated the transcriptional regulations specific to p190-CML in contrast with the default p210-CML. We performed RNA sequencing of diagnostic peripheral blood samples from three p190-CML and three p210-CML patient samples. Although the transcriptional profiles of CML patient samples demonstrated notable clustering according to the Bcr-Abl isoform type (Fig. 1b, Supplementary Fig. 1a), only few genes (*n* = 14) were identified as differentially expressed (DE) and were all upregulated in p190-CML compared to p210-CML samples. Among DE genes were *CCL20, CXCL8 (IL8), IL1R2* (related to cytokine signaling), *IFIT3, IFIT2* (type-I interferon-induced genes), *GPR84* and *S100P* genes (Fig. 1c, Supplementary Fig. 1b). Gene set enrichment analysis revealed significant upregulation of TNF signaling via NFKB, interferon (IFN) alpha and gamma responses, IL1R, apoptosis and P53 pathways in p190-CML in comparison with p210-CML patients (Fig. 1d, Supplementary Fig. 1c, Supplementary Table 3).

Given the reported differences of phospho-proteomic profile between p190 and p210, we next aimed to investigate the phosphorylation levels of key signaling molecules in p190-CML cells. We analyzed samples from three p190-CML and three p210-CML patients. Increased phosphorylation levels of Src and PAK1 kinases were noted in p190 patients. Regarding JAK/STAT signaling, STAT1 and, to lesser extent, JAK1 molecules (related to IFN signaling) showed notable hyperphosphorylation in p190-CML compared to p210-CML samples. Other STAT molecules including STAT3 and STAT5 showed mild hyperphosphorylation in P190-CML samples (Fig. 1e).

### Modeling of Bcr-Abl isoforms in progenitor cell lines highlighted distinct transcriptional regulation and differential signaling

Next, we aimed to get deeper insights of the p190-specific transcriptional and signaling pathways in hematopoietic progenitor cell lines to overcome the limitation of scarcity of p190-CML samples. We used two different models: Ba/f3 cell line, a mouse pro-B that has been extensively used in Bcr-Abl signaling comparative studies [23, 24] and a mouse hematopoietic progenitor cell line, HPC-LSK [25], allowing the characterization of Bcr-Abl isoforms in a progenitor cell model rather than lineage-committed cells.

**Fig. 2:**
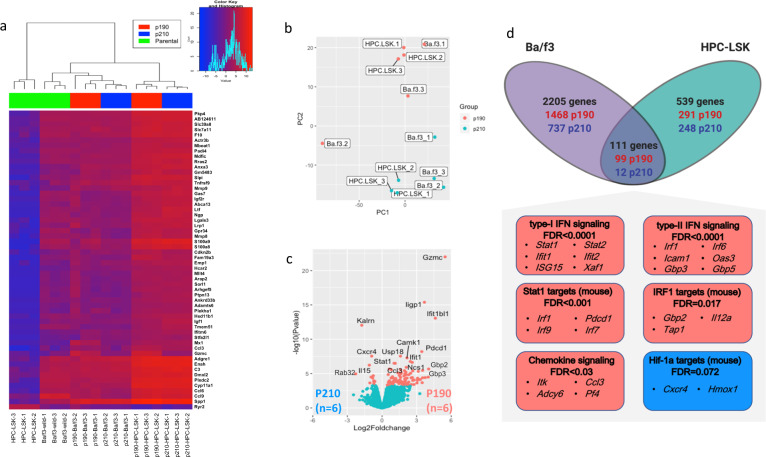
Transcriptional regulations in p190 and p210 cell line models. **a** Heatmap of top 50 variably expressed genes among Ba/f3 and HPC-LSK cell lines (*n* = 12). Fading blue colors indicate down-regulation of the gene in the sample and red its up-regulation relative to the mean expression of the genes across all samples. The Explanatory track indicate the isoform type. Clustering was performed for both genes and samples using the Euclidean distance and Ward linkage method. **b** PCA analysis of transcriptional data (protein coding genes) from Ba/f3 and HPC-LSK cell lines. **c** Volcano plot of protein coding genes between p190 (*n* = 6, right) and p210 (*n* = 6, left) from combined cell lines data. Each gene is represented by a black dot and significantly differentially expressed genes (Q < 0.05, Bayesian statistical test) are colored red. **d** Venn diagram showing the number of differentially expressed genes between p190 and p210 cell lines in Ba/f3 (violet) and HPC-LSK models(blue). 2316 genes and 650 genes were differentially expressed between p190 and p210 in Ba/f3 and HPC-LSK models respectively. Of these genes, 111 were differentially expressed between the two isoforms in both models. Boxes show depiction of the main molecular pathways significantly deregulated between p190 and p210 from the combined data. The color of the boxes as well as numbers in Venn diagram indicate upregulation in p190 (red) or p210 (Blue). Genes enlisted are significantly differentially expressed between p190 and p210 (*Q* < 0.05) in the combined model data. The full list of differentially expressed genes and enriched pathways can be found in Supplementary Table 4.

Transcriptomic comparisons of the cell lines revealed a clear segregation between the parental and p190/p210-transduced cells in both models (Fig. 2a). Despite partly overlapping transcriptional profiles of the isoforms (~70% of differentially expressed genes between either of the isoforms and parental line, i.e., common BCR-ABL1 signature), a notable clustering depending on the isoform type (p190/p210) was observed (Fig. 2b, Supplementary Table 4). In Ba/f3 model 2316 genes were differentially expressed between two isoforms (mostly upregulated in p190 cells), whereas in HPC-LSK cells lower number of genes (650) were found to be differentially expressed (Fig. 2c-d, Supplementary Fig. 2a-b).

To identify high confidence isoform-specific DE genes, we combined data from Ba/f3 and HPC-LSK models after adjusting for possible confounding factors. We identified 111 DE genes between p190 and p210 models (*Q* < 0.05, Fig. 2c-d, Supplementary Table 4). Among p190-overexpressed genes were many IFN-regulatory and response factors, guanylate-binding proteins (*Gbp*), *Stat1* and *Stat2* signaling molecules, lymphocyte-related kinases and markers (*Lck, Lat2, Ly6a*), and cytokine and chemokine molecules. In contrast, the overexpression of *Cxcr4, Il15, Rab32*, and *Kalrn* genes were demonstrated in p210 cells (Fig. 2c, Supplementary Fig. 2c). Pathways enrichment analysis of combined data confirmed upregulation of IFN, chemokines, and apoptosis pathways in addition to *STAT1* and *IRF1* targets in p190 cells compared to *HIF-1a* pathway upregulation in p210 cells (Fig. 2d).

**Fig. 3:**
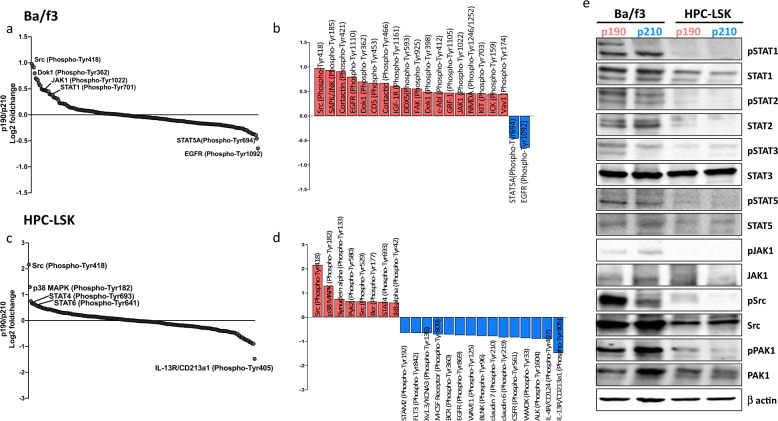
Phospho-signaling profiles of p190 and p210 cell lines. Relative abundance of normalized phospho-proteins signals between p190 and p210 in (**a**) Ba/f3 and (**c**) HPC-LSK cell lines based on phospho-array results (Log2 scale). Signals have been normalized to the median antibody signal of each slide. The top differentially phosphorylated tyrosine-sites proteins in (**b**) Ba/f3 and (**d**) HPC-LSK cell lines between p190 and p210. the cutoff value was set to ±33% in Ba/f3 cells and ±50% in HPC-LSK cells. the color indicates the direction of increased (red) or decreased (blue) phosphorylation in p190/p210 comparison. **e** Western blot analysis of JAK/STAT pathway, Src and PAK1 signaling molecules in cell line models. For each protein a phospho-specific antibody was used in addition to an antibody against the total protein to assess the overall protein expression. Protein quantifications are presented in Supplementary Fig. 3.

In addition to transcriptional programming, we aimed to investigate the p190-specific phosphorylation activity on key signaling molecules in the light of p190-CML patients’ data. First, we investigated the tyrosine phosphorylation profile of p190 and p210 in both models using an extensive array including 228 tyrosine residues (Supplementary Table 5). Src kinase (pTyr418) showed increased phosphorylation in p190 cells in both models (Fig. 3a-d). Regarding JAK/STAT pathway, we measured the phosphorylation levels at tyrosine residue sites essential for STAT dimerization and subsequent nuclear shuttling and transcription activation. STAT6, STAT1, and STAT4 showed increased phosphorylation in p190 in contrast with increased phosphorylation of STAT5 in p210-Ba/f3 cells (Fig. 3a-d). Validation with western blotting demonstrated hyperphosphorylation of STAT1 in p190 models (especially Ba/f3) and to lesser extent STAT2 molecules. STAT3 and STAT5 showed phenotype specific regulation, and in p190-Ba/f3 cells increased phosphorylation was noted, whereas in p190-HPC cells phosphorylation levels were lower, and no difference between isoforms was noted (Fig. 3e, Supplementary Fig. 3). Other signaling molecules including Src and PAK1 exhibited hyperphosphorylation in p190 models in concord with p190-CML data.

### Identification of potential targeted therapies in p190-cells through drug sensitivity profiling

**Fig. 4:**
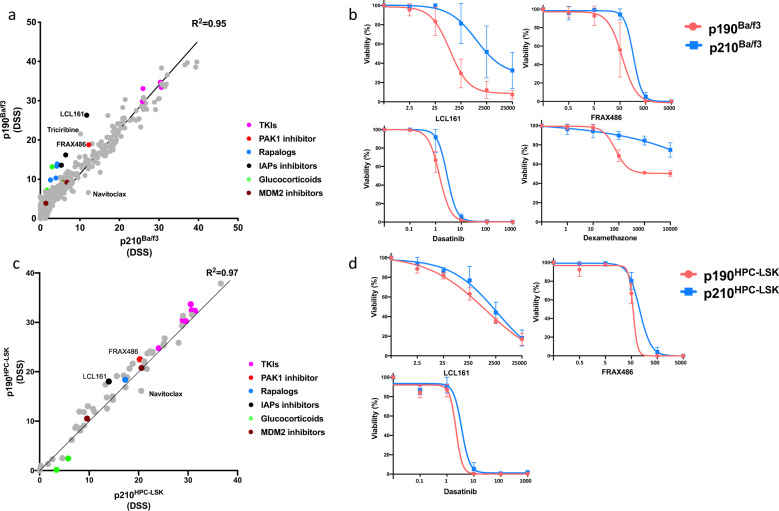
Isoform specific drug sensitivities in cell line models. **a** Scatter plot comparing average drug sensitivity score (DSS) of p190-Ba/f3 and p210-Ba/f3 cells. Drug sensitivity and resistance testing (DSRT) experiments have been performed in triplicates for each setting. Color indicates different drug families (primary targets). **b** Dose response curves showing drug responses of p190-Ba/f3 (red) and p210-Ba/f3 (blue) cell lines to LCL161 (IAP inhibitor), FRAX486 (PAK inhibitor), dasatinib (TKI with Src inhibitory activity) and dexamethasone (glucocorticoids). Concentrations of drugs are indicated in nM. Drug responses to other drugs from the indicated families are available in Supplementary Fig. 4. **c** Scatter plot comparing average drug sensitivity score (DSS) of p190-HPC-LSK and p210-HPC-LSK cells. Drug sensitivity and resistance testing (DSRT) experiments have been performed in triplicates for each setting. Color indicates different drug families (primary targets). **d** Dose response curves showing drug responses of p190-HPC-LSK (red) and p210-HPC-LSK (blue) cell lines to LCL161 (IAP inhibitor), FRAX486 (PAK inhibitor) and dasatinib (TKI with Src inhibitory activity). Concentrations of drugs are indicated in nM.

Next, we aimed to investigate how the transcriptional and signaling differences between p190 and p210 models are reflected in drug sensitivity profiles. For the rapidly proliferating Ba/f3 model, we quantified the drug sensitivities using a comprehensive library of 528 approved and investigational drugs over a 10,000-fold concentration range (Supplementary Table 2). The two isoforms revealed overlapping profiles, and only a few drugs showed preferential sensitivity in p190 cells, including inhibitors of apoptosis (IAP) inhibitors, p21-activating kinase-1 (PAK1) inhibitor, AKT inhibitor, glucocorticoids, and to less extent, rapalogs, MDM2 and JAK inhibitors. In addition, TKIs exerting Src-kinase inhibitory activity (ex: dasatinib, bosutinib, saractinib) exhibited a trend of enhanced sensitivity in p190 cells (Fig. 4a-b, Supplementary Fig. 4a) in accordance with noted Src hyperphosphorylation in p190-CML patient cells and cell line models.

**Fig. 5:**
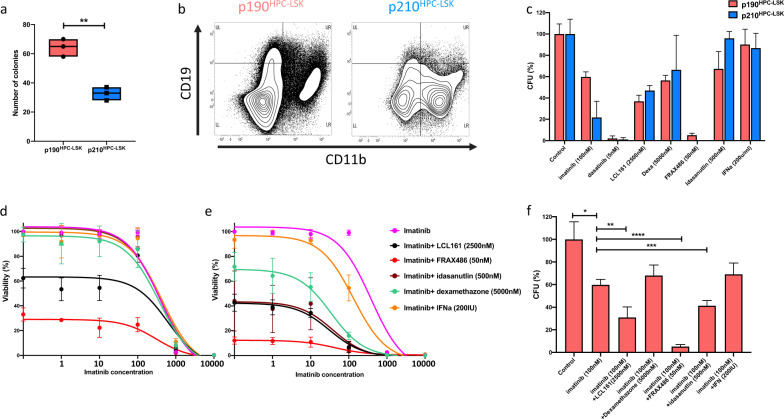
Colonogenic potentials and drug combination strategies in p190 and p210 cell lines. **a** Number of colonies produced by p190-HPC-LSK and p210-HPC-LSK cell lines. **b** Density plot showing phenotypic analysis of harvested cells from p190-HPC-LSK and p210-HPC-LSK colonies using flowcytometry. CD19 (lymphoid marker) on vertical axis and CD11b (granulocytic marker) on horizontal axis. **c** Comparison of the inhibitory effect of imatinib, dasatinib, LCL161, dexamethasone, FRAX486, idasanutlin, and IFNα on colonogenic potentials of p190-HPC-LSK and p210-HPC-LSK. Bar height indicate the average colony counts (normalized to respective control condition) and error bars represent standard deviation. Dose response curves of percent inhibition achieved with imatinib alone and in combination with indicated concentrations of LCL161, FRAX486, idasanutlin, dexamethazone, and IFNα in (**d**) p190-Ba/f3 and (**e**) p190-HPC-LSK cell lines. The experiments were conducted in triplicates. **f** Comparison of the inhibitory effect of imatinib (100 nM) alone and in combination with the indicated concentrations of LCL161, FRAX486, idasanutlin, dexamethazone, and IFNα on the colonogenic potentials of p190-HPC-LSK cells. (*) means *p* < 0.05, (**) means *p* < 0.01, (***) means *p* < 0.001, and (****) means *p* < 0.0001.

Given the p190-specific drug sensitivities in Ba/f3 model, we designed a selected custom panel of 65 drugs for the progenitor HPC-LSK model (Supplementary Table 2). Similar to Ba/f3 cells, LCL-161 (IAP inhibitor) and FRAX486 (PAK inhibitor) exhibited slightly higher activity in p190-HPC-LSK cells in contrast with navitoclax showing more sensitivity in p210-HPC-LSK cells (Fig. 4c-d, Supplementary Fig. 4b). In addition, p190 cells demonstrated increased sensitivity to ex-vivo IFNα treatment compared to p210 cells in both models (Supplementary Fig. 4c). We also compared the effect of selected drugs on the clonogenic potential of p190 and p210 HPC-LSK cells. Despite comparable expression levels of p190 and p210 isoforms (Supplementary Fig. 4d), p190-HPC-LSK cells exhibited higher colony counts and increased differentiation into lymphoid (CD19+) rather than myeloid (CD11b+) phenotypes in contrast with p210 cells (Fig. 5a-b). Similar phenotypic differences were demonstrated in culture where p190-HPC-LSK exhibited more stem-like phenotype with a tendency to differentiate into lymphoid and monocytic lineages, in contrast with granulocytic preference of p210-HPC-LSK cells. IFNα treatment induced expression of Sca-1 (Stem cell antigen-1) and Ly6c (monocyte marker) more strongly in p190 compared to p210 HPC-LSK cells (Supplementary Fig. 5). In addition, p190 cells demonstrated reduced sensitivity to imatinib which was abolished using a TKI with Src inhibitory activity, i.e., dasatinib compared to p210 cells. Furthermore, p190-cells showed increased sensitivity to LCL-161, idasanutlin (MDM2 inhibitor), and dexamethasone (Fig. 5c).

Considering the drug sensitivity profiles of p190 and p210 cells, we investigated whether combining imatinib with other potentially useful drugs would enhance the inhibitory activity in p190 cells. We tested imatinib in combination with LCL-161, FRAX486, idasanutlin, dexamethasone, and IFNα in Ba/f3 and HPC-LSK models (Fig. 5d-e, Supplementary Fig. 4e-f). Interestingly, FRAX486 and LCL-161 consistently enhanced the sensitivity to imatinib in both models. Idasanutlin and dexamethasone revealed variable effects, but in HPC-LSK model combination with imatinib was efficacious with both drugs. IFNα demonstrated a minimal, but consistent, effect in enhancing the sensitivity to imatinib (Fig. 5d-e). Furthermore, the combinations of imatinib with LCL-161, FRAX486 and idasanutlin revealed an enhanced inhibition of the clonogenic potential of p190-HPC-LSK cells (Fig. 5f).

### Validation of isoform-specific phosphorylation and drug sensitivity profiles in primary Ph + ALL cells

Next, we investigated phosphorylation status of selected key signaling molecules including STATs and Src kinase using flow cytometry in samples of a Ph+ALL cohort (*n* = 9). Comparable to p190-CML, p190-Ph+ALL demonstrated increased Src, and to lesser extent STAT5, phosphorylation in contrast with STAT3 that was more phosphorylated in p210 samples (Supplementary Fig. 6a). Finally, we quantified drug responses for selected drugs using 8 Ph+ALL samples (5 p190 and 3 p210 samples). In accordance with p190 cell line models, p190-Ph+ALL primary cells revealed enhanced sensitivity to glucocorticoids and MDM2 inhibitors and to lesser extent to Src-inhibiting TKIs. LCL-161 and FRAX-486 showed similar efficacies in both p190 and p210-Ph+ALL samples (Supplementary Fig. 6b). Testing of drug responses in primary p190 CML cells was not possible due to lack of alive cells which are needed for the assay.

## Discussion

Despite the signaling differences between p190 and p210 Bcr-Abl isoforms have been investigated in cell line models, the knowledge of the differences between these isoforms in primary leukemia cells, especially in CML, is still in infancy. Furthermore, a systematic study of how these differences are reflected on drug sensitivities have never been performed. In this study, we integrated genomic, phosphorylation and drug sensitivity profiling to characterize p190-specific signature in CML in addition to hematopoietic progenitor cell line models. We identified p190-specific upregulation of IFN pathways and activation of the relevant STAT1 and STAT2 in addition to other key signaling molecules including Src and PAK1 kinases. We also identified promising drugs that can be used in combination with TKIs to treat the high-risk p190 patients.

Several clinical studies reported the incidence of p190 in a minority of CML patients, associated with characteristic hematological and clinical features [10, 13]. Previously reported p190-specific features including monocytosis, lack of basophilia and incidence of ACA at diagnosis [14, 15], were also noted in some of p190-CML patients in our study. In addition, p190-CML patients showed inferior response or primary resistance to imatinib frontline treatment, in concord with previous clinical reports [13, 28, 29]. Moreover, genetic profiling revealed that the mutational landscape of p190-CML patients is comparable to the default landscape of chronic phase CML patients with dominance of epigenetic modifier mutations [30, 31]. No *ABL1*- kinase domain mutations were identified in p190-CML patients, suggesting an intrinsic p190 resistance to imatinib.

JAK-STAT signaling is a critical downstream pathway of Bcr-Abl [32]. Differential phosphorylation activity of Bcr-Abl isoforms on STAT molecules has been previously reported in mouse cell lines with increased phosphorylation of STAT5 and STAT3 in p210 and STAT6 and STAT1 in p190 cell lines [4, 20, 24]. Our analysis revealed similar differential phosphorylation of STAT molecules in hematopoietic progenitor cell models. Moreover, we provided the first evidence of p190-specific hyperphosphorylation of STAT1 in CML patients. STAT1 has been reported to be enriched in the interactome of p190, suggesting that STAT1 is a direct target for p190 tyrosine phosphorylation [24]. STAT1 phosphorylation is tightly linked to IFN signaling [33, 34]. In addition, we identified specific upregulation of IFN signaling pathways together with the overexpression of IFN downstream targets. Moreover, p190-HPC-LSK demonstrated enhanced expression of sca-1 and Ly6C which have been linked to IFN-induced STAT1-dependant signaling [35]. The upregulation of IFN signaling together with STAT1 hyperphosphorylation has been previously linked to IFNα treatment sensitivity [36]. In p190 cells, STAT1/STAT3 ratio is shifted toward STAT1-dominant signaling which prime cells for IFNα sensitivity [36, 37]. STAT1 is mediating the antiproliferative activity of therapeutic IFNα [38], and CML cells lacking STAT1 expression have shown to be resistant to IFNα treatment [39]. Our results also demonstrated a consistent, but modest, sensitivity of p190-cell lines to IFNα treatment alone, and it was further enhanced with imatinib combination treatment, which could be an attractive treatment option for further investigation in high-risk p190-CML patients.

Other promising druggable signaling mediators that showed specific association with p190 in both patients and cell line models included Src and PAK1 kinases. Src kinases are enriched in the interactome of p190 compared to p210 in cell line models [24]. Src activity has also been suggested to be essential for p190-induced ALL development [40]. We demonstrated Src hyperphosphorylation in p190 cells of CML and Ph+ALL patients as well as cell line models. Drug sensitivity profiling revealed an enhanced sensitivity of TKIs with Src inhibitory activity in p190 cells, in concord with previously reported efficiency of dasatinib to suppress Ph+ALL better than imatinib [40, 41]. PAK1 is another interesting target, which has been shown to be essential for BCR-ABL1 induced leukemogenesis [42]. In p190-CML cells and cell lines, we demonstrated hyperphosphorylation of PAK1 together with transcriptional upregulation of IL-1B pathway, linked to PAK1 activity [43]. Furthermore, PAK1 inhibitor (FRAX486) showed potent activity in suppressing p190 cells alone and in combination with imatinib. Recently, imatinib combination with PAK1/2 inhibitors showed synergistic effect against CML cells [44].

Targeting apoptosis pathways are potentially promising approaches in the management of leukemias including CML [45, 46]. Our data revealed upregulation of P53 and apoptosis pathways in p190-CML samples and cell lines. Furthermore, we demonstrated increased sensitivity of p190 cells to two classes of apoptotic modulators, IAP inhibitors, and P53 modulators/MDM2 antagonists. IAP inhibitors/SMAC mimetic drugs (ex: LCL161) target the antiapoptotic IAP molecules including cIAP1, cIAP2, and survivin and have shown potential activity in CML as a monotherapy and in combination with TKI [47–49]. Interestingly, p190 has been shown to cooperate with Src activation to drive γ-catenin/MYC-induced survivin overexpression [50], which may partly explain enhanced sensitivity of p190 cells to IAP inhibitors over p210. Moreover, IAP inhibitors upregulate IFNα signaling in tumor cells [51], which can further prime IFNα-upregulated p190 cells for apoptosis. In concord, we demonstrated a significant overexpression of the proapoptotic IFN-induced XAF1, which form a complex with XIAP and induce degradation of IAPs [52].

Transcriptomic regulation in p190 and p210 cells highlighted the differences in phenotypic potentials between the different Bcr-Abl isoforms. Earlier studies have shown that p190 and p210 induce distinct leukemia phenotypes in mouse models [18, 53], In concord, we noted phenotypic preferences of different isoforms in colonogenic assay of HPC-LSK cells, where p190 was associated with stem-like phenotype and increased tendency for lymphoid development in contrast with myeloproliferative phenotype of p210. Furthermore, transcriptional data demonstrated upregulation of several lymphoid markers in p190 cell lines, in contrast to CML-related genes including *Cxcr4* [54] and *Rab32* [55] in p210 models. STAT1-dependant IFN signaling has been shown to induce stemness in cancer cells [56], that might contribute to p190-associated phenotype. In addition, β/γ-catenin switch was recently suggested to contribute to phenotypic fate of Ph+ leukemias, where γ-catenin is critical for p190-ALL development and β-catenin is dominant in p210-CML [50]. In concord, γ-catenin gene (*JUP*) was downregulated in p190-CML (Supplementary Fig. 1b) in contrast with significant upregulation of *GPR84* gene which is tightly linked to aberrant β-catenin activity [57]. In contrast, *Jup* was significantly upregulated in p190 HPC-LSK progenitor cell line model (Supplementary Fig. 2c), where it associates with increased lymphoid differentiation, confirming an essential role of β/γ-catenin in the regulation of phenotypic selection. Cell phenotype can also affect the isoform-specific drug sensitivities. For example, glucocorticoids demonstrated p190-specific activity in Ba/f3 lymphoid cell line as well as in Ph+ALL patients, while they were mostly inactive in HPC-LSK cell line. Taken together, the data suggest that the regulation of phenotypic selection is a complex process and not solely controlled by the isoform type. Transcriptional profiling of additional p190-CML samples is essential to further understand the pathogenic mechanisms controlling BCR-ABL1+ leukemia phenotype.

In summary, our data provides novel insights into the p190-specific genomic and signaling regulation in CML. Our data suggests a critical role of IFN/STAT1 signaling in p190-CML that represents a promising target of treatment. In addition, p190-CML patients typically showing inferior response to imatinib could benefit from frontline dasatinib treatment that additionally targets activated Src signaling. Drug sensitivity profiling also highlighted IAP-, PAK- and MDM2-inhibitors as potentially effective therapeutic options that can be combined with TKIs warranting their testing in personalized treatment strategies of p190-CML.

## Supplementary information

SUPPLEMENTAL MATERIAL

Supplementary Dataset 1

Supplementary Dataset 2

Supplementary Dataset 3

Supplementary Dataset 4

Supplementary Dataset 5
