# Supplementary material for: Characterization of p190-Bcr-Abl chronic myeloid leukemia reveals specific signaling pathways and therapeutic targets
Source: Leukemia. 2020 Nov 9;35(7):1964–75. doi: 10.1038/s41375-020-01082-4 (PMC8257498; doi:10.1038/s41375-020-01082-4)
Supplement: Supplementary file 1 — SUPPLEMENTAL MATERIAL [file 41375_2020_1082_MOESM1_ESM.docx]

**Characterization of p190-Bcr-Abl Chronic Myeloid Leukemia Reveals Specific Signaling Pathways and Therapeutic targets**

**Running title:** p190-Bcr-Abl isoform in CML

Shady Adnan-Awad^1,2,3^, Daehong Kim^1,2^, Helena Hohtari^1,2^, Komal Kumar Javarappa^4^, Tania Brandstoetter^5^, Isabella Mayer^5^, Swapnil Potdar^4^, Caroline A. Heckman^4,6^, Soili Kytölä^7^, Kimmo Porkka^1,2,6^, Eszter Doma^5^, Veronika Sexl^5^, Matti Kankainen^1,2,6^, Satu Mustjoki^1,2,6^

^1^Hematology Research Unit Helsinki, University of Helsinki and Helsinki University Hospital Comprehensive Cancer Center, Helsinki, Finland

^2^Translational Immunology Research program and Department of Clinical Chemistry and Hematology, University of Helsinki, Helsinki, Finland

^3^Clinical pathology department, National Cancer Institute, Cairo University, Cairo, Egypt

^4^ Institute for Molecular Medicine Finland (FIMM), Helsinki Institute for life science, University of Helsinki, Helsinki, Finland

^5^Institute of Pharmacology and Toxicology, University of Veterinary Medicine Vienna, Austria

^6^iCAN Digital Precision Cancer Medicine Flagship.

^7^HUS Diagnostic Center, HUSLAB, Helsinki University Hospital, Helsinki, Finland

**Corresponding author**:

Prof. Satu Mustjoki

Hematology Research Unit Helsinki, University of Helsinki and Helsinki University Hospital Comprehensive Cancer Center, Haartmaninkatu 8, P.O. Box 700, FIN-00290 Helsinki, Finland, Tel +358 9 471 71898, Fax +358 9 471 71897, e-mail: satu.mustjoki@helsinki.fi

**Supplementary Figures**

**Supplementary figure 1.** Transcriptional regulation in p190-CML patients. a) Heatmap of top 50 variably expressed genes among CML patient samples (n=6). Fading blue colors indicate down-regulation of the gene in the sample and red its up-regulation relative to the mean expression of the genes across all samples. The Explanatory track indicate the isoform type. Clustering was performed for both genes and samples using the Euclidean distance and Ward linkage method. b) Comparison of the expression levels (CPM log_2_ values) of *IFIT2, IFIT3, IL1R2, GRP84, TNF, and JUP genes* between p190 and p210 CML samples. Asterisk marks non-significant genes. Other genes have significant Q < 0.05. c) GSEA output showing upregulation of IFN-g and apoptosis pathways in p190-CML patients compared to p210-CML patients.

**Supplementary figure 2.** Transcriptional regulation in p190-cell line models. Volcano plot of protein coding genes between p190 (n=3, right) and p210 (n=3, left) from a) Ba/f3 and b) HPC-LSK cell lines. Each gene is represented by a black dot and significant differentially expressed genes (Q<0.05, Bayesian statistical test) are colored red. c) Comparison of the expression levels (CPM log_2_ values) of the indicated genes between p190 and p210 cell lines.

**Supplementary figure 3.** Quantitative presentation of protein expression levels. Western blot data, presented in figure 3e, were analyzed using ImageJ software (version 2.0.0) to generate quantitative presentation of relative protein expression. Total protein levels were first normalized using respective β-actin levels and represented as relative expression (p190/p210 ratio where p210 values were normalized to one) for a) Ba/f3 and c) HPC-LSK. Phosphorylated protein levels were calculated as a fraction of total protein by dividing the intensities of phosphorylated proteins by the respective total protein intensities for b) Ba/f3 and d) HPC-LSK. The phosphorylated fractions were adjusted for respective total protein level ratios between the two isoforms. JAK1 protein could not be detected in HPC-LSK cell lines.

**Supplementary figure 4.** Drug responses of p190 and p210 cell lines. Comparison of drug responses (DSS scores) between p190 and p210 in a) Ba/f3 and b) HPC-LSK cell lines (n=3, each) of different drug families including TKI with Src inhibitory activity (dasatinib, bosutinib, saractinib), IAP inhibitors (LCL161, Birinapant, AT-406), MDM2 inhibitors (idasanutlin, SAR405838) and JAK inhibitors (ruxolitinib, cerdulatinib, momlotinib/lestaurtinib). Bar height indicates the average DSS score and error bars indicates standard deviation. c) Comparison of responses of p190 and p210 cell lines to recombinant IFNα. The experiment has been performed in triplicates and IFNα concentration are indicated in IU/ml. d) Immunoblot showing the expression levels of p190 and p210 isoforms in p190-and p210-HPC-LSK cells (each in duplicate). Parental HPC-LSK cell line and HPC-LSK cells transduced with an empty vector were used as a control. e) Dose response curves of percent inhibition achieved with imatinib alone and in combination with indicated concentrations of LCL161, FRAX486, idasanutlin, dexamethazone and IFNα in f) p210-Ba/f3 and f) p210-HPC-LSK cell lines. The experiments were conducted in triplicates.

**Supplementary figure 5. Phenotypic characterization of p190 and p210-HPC-LSK cells.** Flowcytometry analysis of p190 and p210-HPC-LSK cells at day 14 culture (5 passages). A panel of CD19 (lymphoid marker), Sca-1 (Stem cell marker), CD11b, Ly6G (Myeloid marker) and Ly6c (monocytic marker) was used to characterize the cells. Plots on the right are showing the effect of IFNα treatment (2 days, 200U/ml) on the phenotype of cells. CD11b expression were not changes with IFNα treatment.

**Supplementary figure 6.** STAT phosphorylation and drug responses of Ph+ALL patients. a) Comparison of phosphorylation levels of Src, p38/MAPK, STAT2, STAT3, STAT4, STAT5, STAT6 and between p190-Ph+ALL and p210-Ph+ALL samples using flowcytometry. The mean fluorescence intensity (MFI) of each read was normalized to the median MFI of p210 reads. The box height indicates 75 percentile of the reads, median MFI of both groups is indicated as a line inside the box and mean is indicated by (+) sign. b) Dose response curves showing drug responses of p190-Ph+ALL (red) and p210-Ph+ALL (blue) cell lines to dexamethasone (glucocorticoids), idasanutlin (MDM2 inhibitor), LCL161 (IAPs inhibitor), FRAX486 (PAK inhibitor), dasatinib and bosutinib (TKI with Src inhibitory activity. Concentrations of drugs are indicated in nM.

**Supplementary Tables:**

**Supplementary Table 1.** Clinicopathological characteristics of p190-CML, p210 CML, and Ph+ALL patients.

**Supplementary Table 2.** Drug libraries used for screening patients and cell line samples**.**

**Supplementary Table 3.** Genomic landscape of p190-CML patients**.**

**Supplementary Table 4.** Transcriptional data of p190 and p210 in cell line models.

**Supplementary Table 5.** Phospho-array data of p190 and p210 cell lines.

**Supplementary methods**

***Antibodies***

Primary antibodies against Src (Clone: 32G6, #2123T), phospho-Src Family (Tyr416) (Clone: D49G4, #6943T), Jak1 (Clone: 6G4, #3344T), phospho-Jak1(Tyr1034/1035) (Clone: D7N4Z, #74129S), Stat2 (Clone: D9J7L, #72604S), Stat1 (#: 9172T), phospho-Stat1 (Tyr701) (Clone: D4A7, #7649T), Stat3 (Clone: 79D7, #4904), phospho-Stat3 (Tyr705) (Clone: D3A7, #9145S), Stat5 (#9363), phospho-Stat5 (Tyr694) (Clone: C71E5, #9314), XIAP (#2042), c-IAP2 (Clone: 58C7, #3130S), c-Abl (#2862S), β-Actin (Clone: 8H10D10, #3700S) were purchased from Cell Signaling Technology and phospho-STAT2 (Tyr690) (#SAB4503836) was purchased from Merck. For HPC-LSK cell line phenotyping, the following antibodies were used CD19-PE (eBioscience, #12-0193-82), CD3-APC (#100236), CD45-APC-Cy7 (# 557659) from Biolegend, and CD11b-PerCP-Cy™5.5 (#561114), CD45R/B220-BV421 (#562922), Ly-6G-PerCP-Cy™5.5 (#560602), Ly-6C-APC-Cy7 (#560596), Ly-6A/E(sca-1)- Alexa Fluor® 647 (#565355) from BD Bioscience and .For detection of phospho-protiens by flowcytometry in Ph+ALL samples, samples were fixed with 1.5% formaldehyde (BD Biosciences) for 15 minutes at room temperature, and permeabilized with 100% ice cold methanol (BD Biosciences) for 30 minutes on ice. Cells were then washed and stained with the following phosphoantibodies: Src (pY418)- Alexa Fluor® 488 (#560095), p38 MAPK (pT180/pY182)- Pacific Blue™ (Clone: 36/p38 (pT180/pY182) (RUO), # 560313), Stat1(pY701)- Pacific Blue™ (Clone:14/P-STAT1 (RUO), #560310), Stat3 (pY705)-PerCP-Cy™5.5 (Clone: 4/P-STAT3 (RUO), #560114), Stat4 (pY693)-Alexa Fluor® 647  (Clone: 38/p-Stat4, #558137), Stat5 (pY694)-Alexa Fluor® 647 (Clone 47/Stat5(pY694) (RUO), #612599), Stat6 (pY641)- PerCP-Cy™5.5 (Clone 18/P-Stat6 (RUO), #561195) (all purchased from BD Biosciences) in addition to STAT2(pY689)-Alexa Fluor® 488 (Clone 1021D, # IC8627G-025) from R&D Systems. Cells were acquired by iQue Screener Plus flow cytometer (Intellicyt) and flow data were analyzed with FlowJo software v10 (Treestar).

***RNA sequencing***

RNA isolation and sample processing were mainly performed as previously described(1). For each cell line model, total RNA was isolated from p190, p210 and parental/control cells (three biological replicates for each condition) using miRNeasy Mini Kit (Qiagen). Quantification of RNA was performed using Qubit RNA kit (Life Technologies) and integrity of the isolated RNA was measured by Agilent Bioanalyzer RNApico chip (Agilent). We used an input of 1.5 µg total RNA to perform ribodepletion of rRNA and further RNA-seq library preparation using ScriptSeq v2™ Complete kit for human/mouse/rat (Illumina) for Ba/f3 cells and Truseq standard total RNA preparation kit (illumina) for HPC-LSK cells. Purification of RNA-sequencing libraries were performed using SPRI beads (Agencourt AMPure XP, Beckman Coulter). Library quality was then evaluated using high Sensitivity chips by Agilent Bioanalyzer (Agilent). Paired-end sequencing of all sequencing libraries (n=18) was performed using Illumina HiSeq technology (HiSeq 2000, Illumina).

***Variant analysis***

WES data was mainly analyzed as previously described(1). Shortly, pre-processing of sequence data were performed using Trimmomatic(2) software and passed paired-end reads were next aligned to human reference genome build 38 (EnsEMBL v82) by BWA-MEM(3). SortSAM was employed to sort Aligned reads by coordinate, and PCR duplicates were marked using the MarkDuplicate module of the Picard toolkit (Broad Institute) with default parameters. Next, we applied Genome Analysis Toolkit (GATK)(4) toolset for variants identification. CrossMap(5) and EnsEMBL chain files was used to convert GATK resource files from GRCh37 to GRCh38. Variant calling was done using GATK somatic short variant best practice (version 3.5), supplemented by estimation

of the cross-sample contamination level and filtering of the 8-oxoguanine and deamination artifacts

by several GATK4 tools (CalculateContamination, CollectSequencingArtifactMetrics, and FilterByOrientationBias). Finally, bcftools(6) was employed for normalization of variant calls. Called variants were further filtered and annotated using the Annovar tool(7) against the RefGene database. First, MuTect2 filters were applied to all samples where variants with a TLOD ≥ 6.3 or a TLOD ≥ 5.0 and supported by five or more independent COSMIC(8) were selected and other variants filtered out. Furthermore, variant data were filtered for by removing synonymous and non-frameshift variants, insertion-deletion (indel) ≥10 bp, variants variants with < 10 supportive reads, variant allele frequency ≤ 5.00%, minor allele frequency ≥ 1% in the EPS and 1KG databases, minor allele frequency ≥ 0.01% in general, Finnish, and Non-European ExAC databases, and Gnomad_Fin database. Variants reported in ≥ ten independent COSMIC hematopoietic tissue samples were recovered.

***Analysis of RNA sequencing data***

RNA-sequencing data was analyzed as earlier described(1). In short, pre-processing of RNA-sequencing data was similar to WES data. Alignment of filtered paired-end reads was performed using STAR(9) supported by EnsEMBL gene models to either human or mouse reference genome build 38. Default settings (2-pass per-sample mapping) were applied to the analysis, except for setting the overhang of the splice junctions to 99. Sorting of the aligned reads and removal of PCR duplicate were performed similar to WES SortSAM and MarkDuplicate module of the Picard toolkit. Feature counts were generated using SubRead(10), and then converted to expression estimates using Trimmed Mean of M-values (TMM) normalisation(11). Low-expressed genomic features with a CPM value ≤1.00 in less than half of samples were filtered out. edgeR(12) software was used to perform differential expression testing. In the statistical testing, comparisons between groups included factors for cell model (in mouse cell lines) and sequencing kit. Identified batches were corrected using the removeBatchEffect function in the package limma(13). Storey’s Q-value for multiple comparisons(14) was used to adjust P‐values. Q ≤0.05 was set as a cutoff value to determine differentially expressed genomic features. For subsequent analysis and visualization, batch-corrected CPM data were used. Clustering of gene expression values was performed for both genes and samples using the Euclidean distance and Ward linkage method. To call variants from RNA sequencing data of Ph+ALL patients, variants were called according to the GATK best practice for calling variants on transcriptome sequencing data. In brief, GATK SplitNCigarReads was used to pre-process data and split mapped reads into exon segments. local indel realignment was performed using GATK IndelRealigner, and recalibration of base qualities was performed using GATK BaseQualityScoreRecalibration. GATK HaplotypeCaller was then employd to call variants according to the best practice recommendations regarding the RNA-seq variant analysis workflow.

***Pathway enrichment analysis***

For CML transcriptional data, pathway enrichment analysis was done using GSEA(15) software (Broad Institute). Pre-ranked GSEA analysis was performed using lists of genes ordered by their log-fold change in the batch-corrected CPM data between p190 and p210 groups. GSEA analysis was performed using default values. False discovery rate (FDR) q <0.1 was set as a cutoff to filter analysis output. For cell line models, Enrichr(16,17) tools were employed to perform pathway analysis. For Enrichr analysis, lists of significantly differentially expressed protein coding genes were used to compare between different conditions (three biological replicates for each cell line condition).

***Drug sensitivity and resistance testing (DSRT)***

DSRT was performed mainly as previously described(18). Briefly, Drugs were p384-microwell plates (Corning) using an acoustic liquid handling device (Echo 550, Labcyte Inc.), in five different concentrations, covering a 10 000-fold concentration range. Initially, Five µl of the indicated culture medium per well were added to dissolve compounds followed by shaking the plates for 10 min. Patients samples were thawed, suspended in Mononuclear Cell Medium (MCM; PromoCell), supplemented with 0.5 μg ml−1 gentamicin and 2.5 μg ml−1 amphotericin B, and cells in a single-cell suspension (10 000 cells in 20 µl per well) were seeded using MultiFlo FX dispenser (BioTek). For cell lines, 2.5x10^3^ cells were resuspended in the indicated media (RPMI for Ba/f3 and IMDM for HPC-LSK) and cells were plated using Multi-Drop Combi peristaltic dispenser (Thermo Scientific) for the 528-drug libraries and MultiFlo FX dispenser (BioTek) for custom libraries. After incubation of the plates for 72 h at 37 °C and 5% CO2, CellTiter-Glo 2.0 reagent (Promega, # G9243) was used to measure cell viability according to the manufacturer’s instructions. Luminescence reads of cell viability were acquired using a Pherastar FS plate reader and were then normalized to 100 mM benzethonium chloride-containing wells (positive control) and DMSO-only wells (negative control). Drug screening data was analyzed using Breeze(19), DSRT data analysis platform (<https://breeze.fimm.fi>) developed at FIMM. Drug responses were quantified using the drug sensitivity score (DSS) model(20).

***Western blot analysis***

Western blot was performed mainly as previously described(21). In brief, cells were washed twice with cold PBS and further lysed in ice-cold RIPA buffer supplemented with 1× protease and phosphatase inhibitor cocktail (Thermo Fisher Scientific, #87786). For removal of cell debris, samples were centrifuged for 10 min at 4 °C, 12,000×g. Total protein concentration was measured with Pierce™ BCA Protein Assay Kit (Thermo Fisher Scientific, #23225) and samples were prepared using Laemmli buffer (Bio-Rad Laboratories). After SDS-PAGE, Trans-Blot® Turbo™ Transfer System (Bio-rad) was used to transfer the proteins into a nitrocellulose membrane (0.2 μm pore-size nitrocellulose, Bio-rad). Primary antibodies (1:1000 dilution) were incubated overnight at 4 °C in the Odyssey blocking buffer (LI-COR Biosciences, #927-70001) containing 0.2% Tween 20. Secondary antibodies (1:15,000 dilution) in the blocking buffer containing 0.2% Tween 20 were then incubated for 2 h at room temperature. Proteins bands were visualized using Odyssey Imaging Systems (LI-COR Biosciences).

***References***

1. Adnan Awad S, Kankainen M, Ojala T, Koskenvesa P, Eldfors S, Ghimire B, et al. Mutation accumulation in cancer genes relates to nonoptimal outcome in chronic myeloid leukemia. Blood Adv. 2020 Feb 11;4(3):546–59.

2. Bolger AM, Lohse M, Usadel B. Trimmomatic: a flexible trimmer for Illumina sequence data. Bioinformatics. 2014 Aug 1;30(15):2114–20.

3. Li H. Aligning sequence reads, clone sequences and assembly contigs with BWA-MEM. arXiv:13033997 [q-bio] [Internet].2013 Mar 16; Available from: http://arxiv.org/abs/1303.3997

4. McKenna A, Hanna M, Banks E, Sivachenko A, Cibulskis K, Kernytsky A, et al. The Genome Analysis Toolkit: A MapReduce framework for analyzing next-generation DNA sequencing data. Genome Res. 2010 Sep;20(9):1297–303.

5. Zhao H, Sun Z, Wang J, Huang H, Kocher J-P, Wang L. CrossMap: a versatile tool for coordinate conversion between genome assemblies. Bioinformatics. 2014 Apr 1;30(7):1006–7.

6. Li H. BFC: correcting Illumina sequencing errors. Bioinformatics. 2015 Sep 1;31(17):2885–7.

7. Wang K, Li M, Hakonarson H. ANNOVAR: functional annotation of genetic variants from high-throughput sequencing data. Nucleic Acids Res. 2010 Sep;38(16):e164.

8. Forbes SA, Beare D, Boutselakis H, Bamford S, Bindal N, Tate J, et al. COSMIC: somatic cancer genetics at high-resolution. Nucleic Acids Res. 2017 Jan 4;45(D1):D777–83.

9. Dobin A, Davis CA, Schlesinger F, Drenkow J, Zaleski C, Jha S, et al. STAR: ultrafast universal RNA-seq aligner. Bioinformatics. 2013 Jan 1;29(1):15–21.

10. Liao Y, Smyth GK, Shi W. The Subread aligner: fast, accurate and scalable read mapping by seed-and-vote. Nucleic Acids Res. 2013 May 1;41(10):e108.

11. Robinson MD, Oshlack A. A scaling normalization method for differential expression analysis of RNA-seq data. Genome Biology. 2010 Mar 2;11:R25.

12. Robinson MD, McCarthy DJ, Smyth GK. edgeR: a Bioconductor package for differential expression analysis of digital gene expression data. Bioinformatics. 2010 Jan 1;26(1):139–40.

13. Ritchie ME, Phipson B, Wu D, Hu Y, Law CW, Shi W, et al. limma powers differential expression analyses for RNA-sequencing and microarray studies. Nucleic Acids Res. 2015 Apr 20;43(7):e47.

14. Storey JD. A Direct Approach to False Discovery Rates. Journal of the Royal Statistical Society Series B (Statistical Methodology). 2002;64(3):479–98.

15. Subramanian A, Tamayo P, Mootha VK, Mukherjee S, Ebert BL, Gillette MA, et al. Gene set enrichment analysis: A knowledge-based approach for interpreting genome-wide expression profiles. PNAS. 2005 Oct 25;102(43):15545–50.

16. Chen EY, Tan CM, Kou Y, Duan Q, Wang Z, Meirelles GV, et al. Enrichr: interactive and collaborative HTML5 gene list enrichment analysis tool. BMC Bioinformatics. 2013 Apr 15;14:128.

17. Kuleshov MV, Jones MR, Rouillard AD, Fernandez NF, Duan Q, Wang Z, et al. Enrichr: a comprehensive gene set enrichment analysis web server 2016 update. Nucleic Acids Res. 2016 Jul 8;44(W1):W90-97.

18. Pemovska T, Kontro M, Yadav B, Edgren H, Eldfors S, Szwajda A, et al. Individualized Systems Medicine Strategy to Tailor Treatments for Patients with Chemorefractory Acute Myeloid Leukemia. Cancer Discov. 2013 Dec 1;3(12):1416–29.

19. Potdar S, Ianevski A, Mpindi J-P, Bychkov D, Fiere C, Ianevski P, et al. Breeze: an integrated quality control and data analysis application for high-throughput drug screening. Bioinformatics. 2020 Jun 1;36(11):3602–4.

20. Yadav B, Pemovska T, Szwajda A, Kulesskiy E, Kontro M, Karjalainen R, et al. Quantitative scoring of differential drug sensitivity for individually optimized anticancer therapies. Scientific Reports. 2014 Jun 5;4:5193.

21. Kim D, Park G, Huuhtanen J, Lundgren S, Khajuria RK, Hurtado AM, et al. Somatic mTOR mutation in clonally expanded T lymphocytes associated with chronic graft versus host disease. Nature Communications. 2020 May 7;11(1):2246.
